# Role of Methylphenidate Augmentation in Treatment-Resistant Obsessive-Compulsive Disorder: A Case Series

**DOI:** 10.7759/cureus.85131

**Published:** 2025-05-31

**Authors:** Varchasvi Mudgal, Saloni Mishra, Upma Gupta, Koustubh R Bagul

**Affiliations:** 1 Psychiatry, Mahatma Gandhi Memorial Medical College, Indore, IND; 2 Physiology, Netaji Subhash Chandra Bose Medical College, Jabalpur, Jabalpur, IND

**Keywords:** dopamine regulation, methylphenidate (mph), neurobiology of ocd, ocd and related disorders, treatment-resistant ocd

## Abstract

Background

Treatment-resistant obsessive-compulsive disorder (tr-OCD) remains a significant clinical challenge, as many patients fail to respond to conventional serotonergic therapies despite multiple augmentation strategies. Emerging evidence from neurobiological research suggests that dopaminergic dysfunction, particularly in the prefrontal cortex, may contribute to the persistence of OCD symptoms. The objective of this study was to evaluate the efficacy and tolerability of methylphenidate (MPH), a dopamine and norepinephrine reuptake inhibitor, as an adjunct in patients with tr-OCD.

Methodology

This case series examines six individuals with tr-OCD who remained symptomatic despite adequate trials of first-line selective serotonin reuptake inhibitors (SSRIs) and various augmentation strategies. MPH was introduced as an adjunct to their existing pharmacotherapy regimens. Clinical outcomes were assessed using the Yale-Brown Obsessive Compulsive Scale (Y-BOCS) at baseline and during follow-up over several weeks, with additional clinical judgment supporting therapeutic decisions.

Results

All six patients demonstrated significant clinical improvement following MPH augmentation. The mean baseline Y-BOCS score was 27.4, which reduced to a mean post-treatment score of 15.1, representing a mean reduction of 12.3 points. Reductions in obsessive thoughts and compulsive behaviors were observed, alongside improvements in cognitive flexibility and impulse control. The treatment was generally well-tolerated, with no serious adverse effects reported.

Conclusion

These preliminary findings suggest that dopaminergic augmentation with MPH may offer therapeutic benefits in cases of tr-OCD. The observed improvements support the hypothesis that enhancing prefrontal dopamine availability can positively influence OCD symptomatology. Further randomized controlled trials are necessary to confirm these results, define optimal dosing strategies, and establish long-term safety and efficacy.

## Introduction

Obsessive-compulsive disorder (OCD) is a debilitating psychiatric condition that significantly impairs an individual’s quality of life. Characterized by persistent intrusive thoughts (obsessions) and repetitive behaviors or mental rituals (compulsions), OCD can lead to severe distress and functional impairment across social, occupational, and personal domains. The global prevalence of OCD is estimated to be around 2-3%, making it a major public health concern. Despite the availability of effective treatments, a substantial proportion of patients fail to achieve adequate symptom relief, leading to what is classified as treatment-resistant OCD (tr-OCD) [1].

Serotonin reuptake inhibitors (SRIs) are considered first-line treatments for OCD. However, many patients remain symptomatic despite an adequate SRI trial. Recently, there has been an emerging literature examining potential ‘‘next step’’ treatments for SRI refractory patients; however, few of these recommendations have been empirically validated [2]. Varied individual response rates to SSRIs, as well as the possible benefit of augmentation therapy with other agents such as antipsychotics, further implicate the role of other neurobiological factors in the pathophysiology of OCD [3].

Tr-OCD is typically defined as the persistence of significant symptoms despite multiple trials of first-line treatments, including high-dose selective serotonin reuptake inhibitors (SSRIs) and augmentation strategies such as atypical antipsychotics, clomipramine, and cognitive-behavioral therapy (CBT) with exposure and response prevention (ERP). Studies suggest that up to 40-60% of patients with OCD do not respond adequately to first-line SSRIs, and approximately 10-20% remain severely impaired despite multiple pharmacological and psychotherapeutic interventions. This treatment resistance imposes a heavy burden not only on affected individuals but also on caregivers and healthcare systems, leading to chronic disability, increased healthcare utilization, and diminished overall well-being [4].

Given the high rates of treatment failure in OCD, there is a critical need for novel pharmacological strategies. In parallel, repetitive transcranial magnetic stimulation (rTMS) has emerged as a promising non-pharmacological intervention, reducing OCD symptoms by modulating activity in brain regions like the dorsolateral prefrontal cortex (PFC) and supplementary motor area [5].

Traditionally, the pathophysiology of OCD has been attributed to dysfunctions in the cortico-striato-thalamo-cortical (CSTC) circuitry, primarily involving serotonergic and glutamatergic systems [6]. However, recent research highlights the role of dopaminergic dysfunction in the disorder, particularly in executive functioning and decision-making processes regulated by the PFC. This growing understanding has opened new avenues for exploring dopamine-modulating agents in the management of OCD [7,8].

This case series aims to explore the potential role of methylphenidate (MPH) as an augmentation strategy for tr-OCD, contributing to the growing body of evidence supporting dopamine-modulating strategies in this disorder. Presenting here are six patients with diverse clinical presentations who showed significant symptom improvement following the addition of MPH to their treatment regimens. These clinical cases, where MPH was used in patients with OCD, provide preliminary insights into its efficacy and tolerability in a subset of patients who remain symptomatic despite conventional treatment approaches. Furthermore, we seek to analyze whether MPH can aid in improving cognitive flexibility and impulse control, which are key deficits in both OCD and tr-OCD. This exploration could pave the way for future research into dopamine-targeted interventions as a viable treatment option for OCD management.

## Materials and methods

This case series was conducted in the Department of Psychiatry at Mahatma Gandhi Memorial Medical College, Indore, with the objective of evaluating the clinical response to MPH augmentation in patients with tr-OCD. Patients who gave consent were recruited using a purposive sampling technique from the outpatient and inpatient services of the department between January 2024 to December 2024.

Inclusion criteria

Patients included in this series met the Diagnostic and Statistical Manual of Mental Disorders, Fifth Edition (DSM-5) diagnostic criteria for OCD and were categorized as having tr-OCD, defined by minimal or no improvement following at least two adequate trials of SSRIs or clomipramine, each administered at the maximum tolerated dose for a minimum of 12 weeks. Additionally, these patients had not responded to at least one augmentation strategy, which could include the addition of an atypical antipsychotic or a sufficient course of CBT incorporating ERP, typically encompassing at least 20 hours of therapist-guided sessions. Patients were required to have a Yale-Brown Obsessive Compulsive Scale (Y-BOCS) score ≥24 at baseline.

Exclusion criteria

Patients with active substance use disorders (other than nicotine), primary psychotic disorders, uncontrolled medical illnesses, or a history of stimulant misuse or movement disorders (such as tics) were excluded. Patients who did not complete the adequate duration of trials with SSRIs due to various reasons, such as lost to follow up, or side effects, were also excluded. Special care was taken to avoid inclusion of individuals with active cases or history of bipolar disorder or schizophrenia, given the potential dopaminergic effects of stimulants.

Clinical assessment and intervention

All patients underwent a comprehensive psychiatric evaluation by qualified psychiatrists. In each case, standard pharmacological regimens had failed to yield satisfactory clinical improvement. Based on emerging evidence implicating dopaminergic dysfunction in OCD, MPH was introduced as an augmentation strategy. The decision to initiate MPH was made collaboratively by the treating team after thorough risk-benefit analysis and discussion with the patient.

MPH was initiated at doses ranging from 30 to 50 mg/day (approximately 0.5 mg/kg/day) in divided doses, using extended-release (ER) formulations. Doses were titrated based on clinical response and tolerability, up to a maximum of 1.2 mg/kg/day. Patients continued their existing psychotropic medications alongside MPH. Follow-up assessments included clinical interviews and Y-BOCS scoring [9] at baseline and during subsequent follow-up visits.

Ethical considerations

Informed consent was obtained from all patients prior to publication of their anonymized clinical data. This case series was conducted in accordance with the ethical standards of the institutional research committee and the 1964 Helsinki Declaration and its later amendments.

## Results

Case 1

A 32-year-old, 68 kg male from rural central India, who operates a small medical shop, presented with a peculiar form of OCD. His primary complaint was an overwhelming fear of being perceived as a coward. To counteract this anxiety, he engaged in prolonged episodes of staring at passersby and even attempted to provoke physical altercations in a bid to “prove” his courage. Notably, he had a history of contamination OCD that had remitted approximately three years earlier following conventional treatment.

After seven months, however, he developed this new obsessional theme, and his symptoms became resistant to multiple treatment strategies. His past interventions included SSRIs such as fluvoxamine 300 mg for 12 weeks, sertraline upto 100 mg for 10 weeks, followed by several augmentation trials with adjunctive agents such as aripiprazole upto 15 mg, risperidone 6 mg, and lamotrigine 100 mg, as well as a trial of clomipramine upto 100 mg, all of which failed to provide satisfactory relief. General physical examination revealed no gross abnormality. On mental status examination, he was well-groomed and cooperative but appeared anxious and preoccupied with his self-image; he demonstrated partial insight into the irrational nature of his behavior. His baseline Y-BOCS score was 26. Given the treatment resistance, his regimen was adjusted to include fluvoxamine 100 mg CR (controlled release) twice a day, and subsequently, after eight weeks, MPH ER was added at a dose of 50 mg in divided doses (30 mg in the morning and 20 mg in the afternoon). After nearly three weeks, he experienced a marked reduction in the frequency and intensity of his compulsive behaviors, and his score improved to 16, indicating a positive response to this augmented treatment approach.

Case 2

A 28-year-old adult male, weighing 72 kg, residing in an urban area and employed at an IT firm, sought help for persistent obsessions centered on indecisiveness. His presenting complaints included an incessant need to ask questions and seek reassurance, which had led to escalating conflicts within his family for four years. His illness had gradually evolved over time, and despite multiple trials with SSRIs (escitalopram 20 mg up to 10 weeks followed by sertraline 150 mg for eight weeks), his symptoms remained refractory, significantly impairing his interpersonal relationships and professional performance. His personal history was notable for a once high-functioning individual, known for his detail-oriented and diligent approach, although recently he had become overwhelmed by his obsessive indecisiveness. General physical examination revealed no gross abnormality. On examination, he was neatly dressed and cooperative, yet his mood was anxious and irritable, and his thought process was dominated by recurrent concerns about decision-making. His baseline Y-BOCS score was 30. Recognizing the limited benefit from conventional therapy, sertraline 150 mg OD (once daily) was augmented with MPH ER of 40 mg OD for six weeks. This led to a significant reduction in his obsessive thoughts and compulsive reassurance-seeking, and his Y-BOCS score reduced to 18 within five weeks of treatment. The patient subsequently reported improved decision-making skills and a notable decrease in familial conflicts, underscoring the potential of dopaminergic augmentation in his case.

Case 3

A 35-year-old married male working as a business trainer, 70 kg by weight, presented with a distressing compulsion: an overwhelming need to divulge every detail of his daily experiences to his friends. This behavior was driven by a pervasive fear that withholding any information would be tantamount to cheating his friends, thereby generating intense feelings of guilt and anxiety. His illness had been resistant to multiple conventional treatment modalities; he had previously undergone rTMS for four weeks, with five sessions per week under OCD protocol, but without success, and experienced erectile dysfunction with SSRIs. Adding to the complexity of his case was a positive family history of bipolar disorder, with his sister being affected, which necessitated a cautious approach in selecting pharmacotherapy. Premorbidly, he had been described as organized, articulate, and responsible, although the emergence of his current symptoms represented a notable departure from his usual behavior. During his mental status examination, he appeared well-dressed and cooperative but was visibly anxious when discussing his compulsive behaviors. His thought process was logical yet overwhelmingly focused on the need to share personal details. To address his refractory symptoms, a combination therapy of clomipramine 100 mg and MPH ER 40 mg OD was initiated, resulting in a substantial reduction in his compulsive disclosure and marked improvements in his social and occupational functioning. His Y-BOCS score improved from 24 to 13 within three weeks of treatment, adding an objective perspective to his improvement.

Case 4

An adult male, known to be a regular smoker and with a significant family history of OCD (his mother had been affected by contamination OCD), presented with highly distressing blasphemous obsessions for six years. He reported that these intrusive thoughts, which were culturally taboo and deeply upsetting, had persisted despite multiple attempts at standard pharmacological treatment. His compulsions and obsessive thoughts severely interfered with his daily activities and social interactions, leading to significant functional impairment. His personal history revealed no major deviations until the onset of these symptoms, which dramatically altered his baseline functioning. No significant abnormalities in physical examination. On mental status examination, he appeared anxious and preoccupied; his mood was markedly anxious, and while his thought process was coherent, it was dominated by intrusive, blasphemous content. His Y-BOCS score was 28. Despite trials with various conventional agents, his symptoms remained intractable. Consequently, his treatment was modified to include a multi-modal approach consisting of fluvoxamine 100 mg OD combined with lamotrigine 100 mg OD, with MPH 40 mg ER OD added as an augmenting agent. This comprehensive regimen led to considerable alleviation of his obsessive thoughts and a reduction in his compulsive behaviors, with Y-BOCS score improving to 14 within four weeks of treatment, highlighting the potential role of MPH in managing complex, tr-OCD cases.

Case 5

A 29-year-old unmarried male working as a civil engineer presented with intrusive symmetry obsessions and compulsive ordering rituals for 10 years. He described a longstanding preoccupation with the need for objects in his environment to be perfectly aligned and symmetrical. Any disruption in symmetry would provoke intense anxiety and a compulsive need to rearrange items until a subjective sense of "rightness" was achieved. These symptoms had begun in late adolescence and had progressively worsened, resulting in significant delays in daily functioning, frequent tardiness at work, and social withdrawal. He had previously undergone multiple adequate trials of SSRIs, including fluoxetine (up to 80 mg/day) and escitalopram (up to 30 mg/day), without meaningful improvement. Augmentation with risperidone 4 mg and clomipramine 75 mg had yielded a partial response but was discontinued due to side effects, including sedation and sexual dysfunction. CBT with ERP had been initiated, and five sessions were conducted for five weeks, but poorly adhered to them due to the patient's cognitive rigidity and poor distress tolerance. On mental status examination, he was alert, cooperative, and oriented but appeared tense and preoccupied with the symmetry of objects in the clinician’s office. He demonstrated good insight into the irrational nature of his behaviors but reported being unable to resist the compulsions. His baseline Y-BOCS score was 27. Given the limited response to standard treatments and the prominent executive dysfunction, MPH was initiated at 20 mg/day and gradually titrated to 40 mg/day with OD dosing. He was maintained on a stable dose of fluoxetine 60 mg/day. Within four weeks, he reported a noticeable reduction in anxiety related to minor asymmetries, and he was increasingly able to delay or resist compulsions. His therapist noted improved engagement with ERP tasks. At six weeks, his Y-BOCS score had decreased to 16, and he expressed confidence in managing his symptoms, allowing him to resume social activities and improve work attendance.

Case 6

A 38-year-old married male, 64 kg by weight, working as an accountant in a metropolitan city, presented with obsessive doubts about whether he had committed minor moral infractions (e.g., accidentally lying, cheating, or offending others unknowingly) for six years. This led to excessive confession rituals and reassurance-seeking from his spouse and colleagues, severely impairing his occupational and social functioning. His history revealed longstanding perfectionistic and guilt-prone personality traits. Despite adequate trials of fluoxetine (up to 80 mg/day) and clomipramine (up to 200 mg/day), augmented with risperidone up to 4 mg and lamotrigine up to 75 mg at different stages, his symptoms remained debilitating. CBT-ERP for seven sessions, two sessions per week for four weeks, yielded only temporary relief, decreasing his anxiety and restlessness, but the distress continued primarily due to pronounced indecisiveness and difficulty tolerating uncertainty.

On examination, he was neatly dressed, tense, and tearful while describing the burden of constant confessions. He exhibited good insight but high anxiety levels, with a baseline Y-BOCS score of 31. His general physical examination revealed no abnormality. Given his executive dysfunction (especially intolerance of uncertainty and impaired impulse control) and refractory nature of symptoms, MPH ER was added at 30 mg/day OD, while continuing fluoxetine at 60 mg/day OD and low-dose aripiprazole 5 mg/day at night. Within three weeks, his frequency of confession rituals reduced, and he reported improved cognitive clarity and impulse control. By the sixth week, his Y-BOCS score dropped to 18. He resumed ERP sessions with renewed willingness, and his spouse reported reduced interpersonal friction. Mild transient insomnia was noted initially, which was managed by an earlier intake of medications.

The clinical profiles, treatment histories, MPH dosages, and outcomes of all six patients are summarized in Table 1. All patients demonstrated varying degrees of improvement in their Y-BOCS scores following MPH augmentation, highlighting its potential utility in tr-OCD.

**Table 1:** Clinical Characteristics and Treatment Outcomes in Patients With Treatment-Resistant OCD Receiving Methylphenidate Augmentation This table summarizes the demographic details, primary obsessive-compulsive themes, prior treatment history, methylphenidate (MPH) dosing, and clinical outcomes of six patients diagnosed with treatment-resistant obsessive-compulsive disorder (tr-OCD). All patients received MPH as an augmentation strategy following inadequate response to conventional therapies. Symptom severity was assessed using the Yale-Brown Obsessive Compulsive Scale (YBOCS). rTMS: repetitive transcranial magnetic stimulation; CBT-ERP: cognitive-behavioral therapy-exposure and response prevention; ER: extended release; CR: controlled release

Case	Age/Sex/Occupation	OCD Symptom Profile	Prior Treatments	Methylphenidate Regimen	Adjunctive Medications	Y-BOCS (Pre)	Y-BOCS (Post)	Outcome
1	32/M, Medical Shop Owner	Fear of being perceived as a coward; staring/provoking fights	Fluvoxamine 300 mg, sertraline 100 mg, clomipramine 100 mg, aripiprazole 15 mg, risperidone 4 mg, lamotrigine 100 mg	50 mg/day ER	Fluvoxamine 200 mg CR	26	16	Marked reduction in compulsions, improved impulse control
2	28/M, IT Professional	Obsessive indecisiveness, reassurance-seeking	Escitalopram 20 mg, sertraline 150 mg	40 mg/day	Sertraline 150 mg	30	18	Improved decision-making, reduced familial conflict
3	35/M, Business Trainer	Compulsive self-disclosure driven by guilt	Sertraline 100 mg, escitalopram 40 mg, clomipramine 100 mg, rTMS	40 mg/day	Clomipramine 100 mg	24	13	Decreased compulsive disclosure, better social/occupational functioning
4	34/M, Smoker	Blasphemous intrusive thoughts	Fluvoxamine 300 mg, sertraline 100 mg, clomipramine 100 mg, aripiprazole 15 mg, risperidone 4 mg, lamotrigine 100 mg	40 mg/day	Fluvoxamine 100 mg+ lamotrigine 100 mg	28	14	Alleviation of intrusive thoughts, better social functioning
5	29/M, Civil Engineer	Symmetry obsessions, ordering compulsions	Fluoxetine 80 mg, escitalopram 30 mg, risperidone 4 mg, clomipramine 75 mg, CBT-ERP 5 sessions, 1 per week (poor adherence)	20-40 mg/day	Fluoxetine 60 mg	27	16	Reduced anxiety, improved ERP engagement, and work performance
6	38/M, Accountant	Obsessive moral scrupulosity, confession rituals	Fluoxetine 80 mg, clomipramine 200 mg, risperidone 4 mg, lamotrigine 100 mg, CBT 7 sessions, 2 per week	30 mg/day	Fluoxetine 60 mg + aripiprazole 5 mg	31	18	Reduced confessions, better uncertainty tolerance, ERP resumed

## Discussion

OCD and attention-deficit hyperactivity disorder (ADHD) are both neuropsychiatric conditions that, despite their distinct clinical presentations, share overlapping features, particularly in the domains of impulse control and executive functioning. OCD is marked by persistent, intrusive thoughts and repetitive behaviors, whereas ADHD is characterized by impulsivity, hyperactivity, and inattention. Importantly, both disorders involve dysfunction within the CSTC. In OCD, excessive activity within these circuits is thought to lead to the reinforcement of compulsive behaviors, while in ADHD, reduced activity contributes to poor inhibitory control [10].

MPH, a dopamine reuptake inhibitor, is widely used in treating ADHD in children and adolescents [11]. Its therapeutic effects in ADHD are primarily mediated by inhibition of the presynaptic dopamine transporter, with a secondary effect on the noradrenaline transporter [12,13]. By increasing the residence time of dopamine in the synaptic cleft, MPH amplifies neurotransmission [12,14]. Alternatively, it has been proposed that MPH may attenuate dopaminergic tone by stimulating presynaptic inhibitory autoreceptors [3].

Recently, MPH has emerged as a potential augmentation strategy in tr-OCD. Preliminary data suggest that MPH can decrease checking compulsions - symptoms that are often comorbid with ADHD [15,16]. Additionally, a case series reported that adjunctive MPH ER reduced both self-reported and objective measures of inattention and hoarding symptoms, which may also benefit patients with OCD [17,18]. Its mechanism involves inhibiting the reuptake of dopamine and norepinephrine, thereby increasing their availability in the synaptic cleft. While conventional perspectives have suggested that excessive dopaminergic activity worsens compulsive behaviors, emerging evidence indicates that selective dopaminergic enhancement in the PFC may improve cognitive flexibility, impulse control, and decision-making functions often impaired in OCD. In doing so, MPH could reduce the intensity of obsessive thoughts and compulsive rituals, offering a novel therapeutic approach for patients who have exhausted standard treatment options [3,18,19]. A sparse body of literature has suggested a potential role for MPH as a treatment option. Compared to Zheng et al. (2019), which showed significant Y-BOCS reduction with MPH in tr-OCD over a structured four-week randomized controlled trial (RCT), the current article offers only anecdotal, non-standardized data with variable follow-ups [3].

Although most first-line pharmacotherapy for OCD targets serotonergic systems, convergent evidence now suggests that dopamine dysregulation may also contribute to OCD’s pathophysiology [7]. Disruption within the CSTC - an area where dopamine transmission is crucial - has been documented in several studies [20,21]. Furthermore, imaging studies have revealed a complex imbalance in the dopamine system among OCD patients [22,23]. The patients in our case series - Cases 1 through 6 - demonstrated significant improvement in obsessive-compulsive symptoms after the introduction of MPH, which could be attributed to its ability to enhance frontal cortical activation [14]. This enhanced cognitive control may have allowed patients to better challenge dysfunctional obsessive beliefs and engage more effectively in therapy that reduced their preoccupation with obsessional content.

This case series highlights the potential utility of MPH augmentation in the management of tr-OCD. Across all six cases presented - varying widely in symptomatology, psychosocial background, and prior treatment failures - the introduction of MPH yielded clinically meaningful improvements. For instance, Case 1, who presented with obsessions about being perceived as a coward and engaged in provocative behaviors, showed marked improvement after MPH augmentation following failure of multiple prior augmentation strategies. Case 2, whose illness was dominated by indecisiveness and compulsive reassurance-seeking, reported substantial gains in decision-making capacity and interpersonal functioning. Case 3, with a compulsion to disclose every detail of his daily life, demonstrated a reduction in compulsive urges and restored occupational functioning, despite a family history of bipolar disorder that had complicated prior treatment. Case 4, who experienced severe blasphemous intrusive thoughts and significant functional impairment, also showed notable symptom relief with MPH augmentation in a multimodal regimen. Case 5, who had symmetry obsessions and ordering rituals with pronounced cognitive rigidity and poor adherence to ERP, achieved improved cognitive flexibility, reduced symptoms, and greater participation in behavioral therapy following MPH introduction. Case 6, presenting with intrusive harm obsessions and compulsive checking, demonstrated significant symptom reduction and improved distress tolerance following MPH augmentation after multiple failed serotonergic and antipsychotic trials. His occupational functioning and ERP engagement markedly improved.

While SRIs remain the gold standard in OCD treatment, a substantial subset of patients remains resistant to these interventions. Augmentation with atypical antipsychotics such as risperidone and aripiprazole has been explored, but response rates remain variable, and side effects such as metabolic disturbances and sedation often limit their long-term use. Alternative strategies, such as glutamatergic agents (e.g., memantine, N-acetylcysteine), rTMS, and deep brain stimulation, are under investigation but are not yet widely implemented in clinical practice. In this context, MPH augmentation presents a promising alternative that is relatively well-tolerated and may yield faster symptom relief in select cases in comparison to other dopaminergic agents with minimal side effects [24].

Given the heterogeneity of OCD, a personalized approach to treatment may improve outcomes. The response to MPH in our cases suggests that specific patient subtypes - particularly those with executive dysfunction, cognitive rigidity, or comorbid ADHD traits - might benefit more from dopaminergic augmentation. For example, Case 5, who initially struggled with ERP due to cognitive rigidity, experienced substantial improvement in both symptom severity and therapeutic engagement after starting MPH. This effect was particularly evident as the patient showed improved motivation and flexibility during ERP sessions, leading to better outcomes than seen with previous treatment approaches. This suggests that MPH may not only reduce core OCD symptoms but also enhance responsiveness to psychological interventions. Future studies should explore potential biomarkers, including genetic and neuropsychological profiles, to identify individuals most likely to respond favorably to MPH augmentation [22].

CBT, particularly ERP, remains a cornerstone in OCD management. Patients with executive dysfunction often struggle with the cognitive flexibility required for successful ERP [25]. By enhancing cognitive control and reducing compulsivity, MPH may facilitate greater engagement with ERP and improve overall treatment efficacy. In Case 5, this effect was particularly evident, as the patient showed improved motivation and flexibility during ERP sessions, leading to better outcomes than seen with previous treatment approaches.

Although MPH is well-studied in ADHD, its long-term use in OCD remains relatively unexplored. Potential concerns include risk of addiction, exacerbation of anxiety symptoms, induction of tics, and the possibility of worsening impulsivity in predisposed individuals [26]. However, none of the patients in our case series reported significant side effects, suggesting that with proper patient selection and monitoring, MPH can be a safe augmentation strategy in tr-OCD.

There are a few limitations to this research. First, this is a small, uncontrolled case series without placebo control, which limits its generalizability. Additionally, the follow-up duration was relatively short, making it difficult to assess long-term remission rates. Given that OCD symptoms often fluctuate, it is unclear whether the observed improvements with MPH will be sustained over extended periods. Also, the lack of standardized anxiety rating scales, such as the Generalized Anxiety Disorder 7-item (GAD-7) scale, prior to and following MPH initiation limits the objective assessment of anxiety-related adverse effects, particularly in an OCD population inherently predisposed to internalizing symptoms. Furthermore, a placebo-controlled trial is needed to determine whether the benefits of MPH are due to its specific pharmacological action or non-specific effects such as increased motivation and arousal.

Despite these limitations, our findings align with prior research suggesting that stimulant medications may hold therapeutic potential in tr-OCD. A double-blind study previously reported that D-amphetamine and caffeine augmentation led to symptom improvement in tr-OCD patients, with responders showing a mean Y-BOCS reduction of 48-55% [27]. These findings add to this emerging literature by highlighting the potential of MPH ER in patients who have failed conventional augmentation strategies.

This case series supports the potential utility of MPH as an augmentation strategy in tr-OCD. The rapid improvement observed in all six patients suggests that dopaminergic modulation, particularly through selective enhancement of PFC function, may alleviate executive dysfunction and reduce compulsive behaviors. While conventional perspectives have linked increased dopamine activity with worsened OCD symptoms, emerging evidence suggests that targeted enhancement of dopaminergic neurotransmission may, paradoxically, improve cognitive control and reduce obsessional severity. Moving forward, RCTs with longer follow-up periods are essential to validate the efficacy and safety of MPH augmentation in tr-OCD. If confirmed, MPH could represent a valuable addition to the armamentarium of treatment options for individuals struggling with persistent, disabling OCD symptoms.

## Conclusions

This case series highlights the potential role of MPH as an effective augmentation strategy in tr-OCD. Across six diverse clinical presentations, MPH was associated with significant reductions in obsessive-compulsive symptoms, particularly in patients with executive dysfunction and poor response to conventional treatments. These findings support the hypothesis that targeted dopaminergic modulation may enhance cognitive flexibility, improve engagement with therapy, and alleviate core OCD symptoms. Further controlled studies are warranted to validate these observations and establish long-term safety and efficacy.

## References

[1] Kayser RR (2020). Pharmacotherapy for treatment-resistant obsessive-compulsive disorder. J Clin Psychiatry.

[2] Pittenger C (2021). Pharmacotherapeutic strategies and new targets in OCD. Curr Top Behav Neurosci.

[3] Zheng H, Jia F, Han H (2019). Combined fluvoxamine and extended-release methylphenidate improved treatment response compared to fluvoxamine alone in patients with treatment-refractory obsessive-compulsive disorder: a randomized double-blind, placebo-controlled study. Eur Neuropsychopharmacol.

[4] Koo MS, Kim EJ, Roh D, Kim CH (2010). Role of dopamine in the pathophysiology and treatment of obsessive-compulsive disorder. Expert Rev Neurother.

[5] Steuber ER, McGuire JF (2023). A meta-analysis of transcranial magnetic stimulation in obsessive-compulsive disorder. Biol Psychiatry Cogn Neurosci Neuroimaging.

[6] Brock H, Rizvi A, Hany M (2024). Obsessive-Compulsive Disorder. https://www.ncbi.nlm.nih.gov/books/NBK553162/.

[7] Milad MR, Rauch SL (2012). Obsessive-compulsive disorder: beyond segregated cortico-striatal pathways. Trends Cogn Sci.

[8] Brem S, Grünblatt E, Drechsler R, Riederer P, Walitza S (2014). The neurobiological link between OCD and ADHD. Atten Defic Hyperact Disord.

[9] Goodman WK, Price LH, Rasmussen SA (1989). The Yale-Brown Obsessive Compulsive Scale. I. Development, use, and reliability. Arch Gen Psychiatry.

[10] Brault MC, Lacourse É (2012). Prevalence of prescribed attention-deficit hyperactivity disorder medications and diagnosis among Canadian preschoolers and school-age children: 1994-2007. Can J Psychiatry.

[11] van Roessel PJ, Grassi G, Aboujaoude EN, Menchón JM, Van Ameringen M, Rodríguez CI (2023). Treatment-resistant OCD: pharmacotherapies in adults. Compr Psychiatry.

[12] Jaeschke RR, Sujkowska E, Sowa-Kućma M (2021). Methylphenidate for attention-deficit/hyperactivity disorder in adults: a narrative review. Psychopharmacology (Berl).

[13] Gamo NJ, Wang M, Arnsten AF (2010). Methylphenidate and atomoxetine enhance prefrontal function through α2-adrenergic and dopamine D1 receptors. J Am Acad Child Adolesc Psychiatry.

[14] German CL, Baladi MG, McFadden LM, Hanson GR, Fleckenstein AE (2015). Regulation of the dopamine and vesicular monoamine transporters: pharmacological targets and implications for disease. Pharmacol Rev.

[15] Gürkan K, Bilgiç A, Türkoglu S, Kiliç BG, Aysev A, Uslu R (2010). Depression, anxiety and obsessive-compulsive symptoms and quality of life in children with attention-deficit hyperactivity disorder (ADHD) during three-month methylphenidate treatment. J Psychopharmacol.

[16] Dogan-Sander E, Strauß M (2021). Case report: treatment of a comorbid attention deficit hyperactivity disorder and obsessive-compulsive disorder with psychostimulants. Front Psychiatry.

[17] Rodriguez CI, Bender J Jr, Morrison S, Mehendru R, Tolin D, Simpson HB (2013). Does extended release methylphenidate help adults with hoarding disorder?: a case series. J Clin Psychopharmacol.

[18] King J, Dowling N, Leow F (2017). Methylphenidate in the treatment of an adolescent female with obsessive-compulsive disorder and attention deficit hyperactivity disorder: a case report. Australas Psychiatry.

[19] Woolley JB, Heyman I (2003). Dexamphetamine for obsessive-compulsive disorder. Am J Psychiatry.

[20] Denys D, de Vries F, Cath D (2013). Dopaminergic activity in Tourette syndrome and obsessive-compulsive disorder. Eur Neuropsychopharmacol.

[21] Pauls DL, Abramovitch A, Rauch SL, Geller DA (2014). Obsessive-compulsive disorder: an integrative genetic and neurobiological perspective. Nat Rev Neurosci.

[22] Perani D, Garibotto V, Gorini A (2008). In vivo PET study of 5HT(2A) serotonin and D(2) dopamine dysfunction in drug-naive obsessive-compulsive disorder. Neuroimage.

[23] Sesia T, Bizup B, Grace AA (2013). Evaluation of animal models of obsessive-compulsive disorder: correlation with phasic dopamine neuron activity. Int J Neuropsychopharmacol.

[24] Koran LM, Aboujaoude E (2018). Promising treatments for obsessive-compulsive disorder: a call for additional research. Curr Med Chem.

[25] McKay D, Sookman D, Neziroglu F (2015). Efficacy of cognitive-behavioral therapy for obsessive-compulsive disorder. Psychiatry Res.

[26] Çoban A, Tan O (2020). Attention deficit hyperactivity disorder, impulsivity, anxiety, and depression symptoms mediating the relationship between childhood trauma and symptoms severity of obsessive-compulsive disorder. Noro Psikiyatr Ars.

[27] Koran LM, Aboujaoude E, Gamel NN (2009). Double-blind study of dextroamphetamine versus caffeine augmentation for treatment-resistant obsessive-compulsive disorder. J Clin Psychiatry.

